# An Electrochemical Aptasensor for Pb^2+^ Detection Based on Metal–Organic-Framework-Derived Hybrid Carbon

**DOI:** 10.3390/bios11010001

**Published:** 2020-12-22

**Authors:** Jina Ding, Dongwei Zhang, Yang Liu, Xuejia Zhan, Yitong Lu, Pei Zhou, Dan Zhang

**Affiliations:** 1 School of Agriculture and Biology, Shanghai Jiaotong University, Shanghai 200240, China; jnding@sjtu.edu.cn (J.D.); Donaghy-Zhang@sjtu.edu.cn (D.Z.); Kimi1201@sjtu.edu.cn (Y.L.); xjzhan@sjtu.edu.cn (X.Z.); ytlu@sjtu.edu.cn (Y.L.); zhdsjtu@sjtu.edu.cn (D.Z.); 2 Key Laboratory of Urban Agriculture, Ministry of Agriculture and Rural Affairs of the People’s Republic of China, Shanghai Jiao Tong University, Shanghai 200240, China

**Keywords:** electrochemical aptasensor, lead, aptamer, nanoporous carbon

## Abstract

A new double-shelled carbon nanocages material was synthesized and developed an aptasensor for determining Pb^2+^ in aqueous solution. Herein, nanoporous carbon materials derived from core–shell zeolitic imidazolate frameworks (ZIFs) demonstrated excellent electrochemical activity, stability, and high specificity surface area, consequently resulting in the strong binding with aptamers. The aptamer strands would be induced to form G-quadruplex structure when Pb^2+^ was introduced. Under optimal conditions, the aptasensor exhibited a good linear relationship of Pb^2+^ concentration ranging from 0.1 to 10 μg L^−1^ with the detection limits of 0.096 μg L^−1^. The feasibility was proved by detecting Pb^2+^ in spiked water samples and polluted soil digestion solution. The proposed aptasensor showed excellent selectivity and reproducibility, indicating promising applications in environmental monitoring.

## 1. Introduction

Heavy metals are distributed worldwide and are harmful to human health and eco-environments [1,2,3,4]. In humans, lead is one of the most common pollutants accumulated through the food chain, thereby damaging human health even at a low concentration [2,5]. Lead ion (Pb^2+^) may cause a serial damaging to human health, such as memory loss, anemia, and irritability [6,7,8]. Therefore, development of new, accurate, and simple methods for Pb^2+^ determination is an urgent task.

In recent years, the sensors based on aptamers have shown great potential in detecting heavy metal ions. Aptamers are short and specific single-strand oligonucleotides, selected through systematic evolution of ligands by exponential enrichment (SELEX) [9,10]. The Pb^2+^-aptamer is a guanine (G)-rich oligonucleotides strand which could form G-quadruplex structure on interacting with Pb^2+^ [11,12,13]. Based on the high affinity and selectivity of aptamers, numbers of aptasensors have been developed for Pb^2+^ detection, such as fluorescent [14,15], colorimetric, chemiluminescent [16,17], and electrochemical analysis [18,19]. 

Electrochemical sensor has been identified as one of the most feasible methods for the detection of heavy metal, for the devices are not only low-cost and relatively simple but have also high selectivity and sensitivity [20,21]. Moreover, a series of nanomaterials have been used in analytical electrochemistry, including gold nanoparticles [22,23,24], metal or metal oxide nanoparticles [25,26], quantum dots [11], graphene [14,27], and metal–organic frameworks (MOFs) [28,29]. MOFs, porous inorganic–organic hybrid materials assembled from metal ions and organic ligands, have attracted attention as a novel material with well-defined porosities, high surface areas, and chemical stability [30,31]. Owing to the excellent characteristics, various biosensors based on MOFs have been developed for detecting heavy metals [29,32], antibiotics [33,34], phosphoprotein [35], and so on. However, MOFs also have some disadvantages, like the non-conducting of MOFs, as well as the unstable bond of inorganic metal ions and organic ligands. Therefore, the research of MOFs gradually transfer from the MOFs to MOF-based derivatives. Derivatives of MOFs, including metal/metal oxide nanoparticles [36], carbon−metal/metal oxide hybrids [37], and porous carbon [38] can be obtained through the solvothermal method and high-temperature calcination method [39]. Metal/metal oxide and carbon nanoparticles have been revealed excellent electrocatalytic activity for the oxygen reduction reaction. However, few studies about MOFs derivatives have been done on the electrochemical sensors. 

In this work, a facile strategy on preparing carbon material (CZIFs) derived from the core–shell zeolite imidazole frameworks (ZIF8@ZIF67) was reported. The CZIFs are carbon-based hybrid double-shelled structure of outer shells of Co-graphitic carbon and inner shells of microporous carbon. The CZIFs and aptamer can generate high immobilization force for the aptamer strands. Owing to this, a new aptasensor was built to detect Pb^2+^ of river water and polluted soil.

## 2. Materials and Methods

### 2.1. Materials and Apparatus

The lead-aptamer (Apt) 5′-CAACGGTGGGTGTGGTTGG-3′ was synthesized by Sangon Biotechnology (Shanghai, China). PbCl_2_ was bought from Merck Co., Inc. (Darmstadt, Germany) and the other reagents were purchased from Shanghai Sinopharm Chemical ReagentCo., Ltd. (Shanghai, China) without further purification. 

Material characterization scanning electron microscope (SEM), transmission electron microscopy (TEM), and XPS analysis were conducted on Hitachi S4800 microscopy, TF20 (FEI) microscopy, and Thermo ESCALAB 250XI, respectively. An electrochemical workstation (CHI1030A, Shanghai Chenhua Instruments, Shanghai, China) was used to measure the current. The screen printed carbon electrode (SPCE) was used in this aptasensor. The SPCE is a three electrode system including a carbon working electrode, an Ag/AgCl reference electrode, and a carbon auxiliary electrode. The full automatic microwave (Auto Digiblock S60 UP, Lab Tech) was employed to digest the polluted soil and the graphite furnace atomic absorption spectrometry (GFAAS, AA900, PE, Winter Street Waltham, MA, USA) was applied to measure the content of Pb^2+^.

### 2.2. The synthesis of CZIFs Particles

ZIF-8 particle was assembled according to previous reports [39]. Furthermore, the detailed preparation processes of the CZIFs material is shown in the Supplementary Information (Figures S1 and S2).

### 2.3. Preparation of Probe

**Scheme 1:**
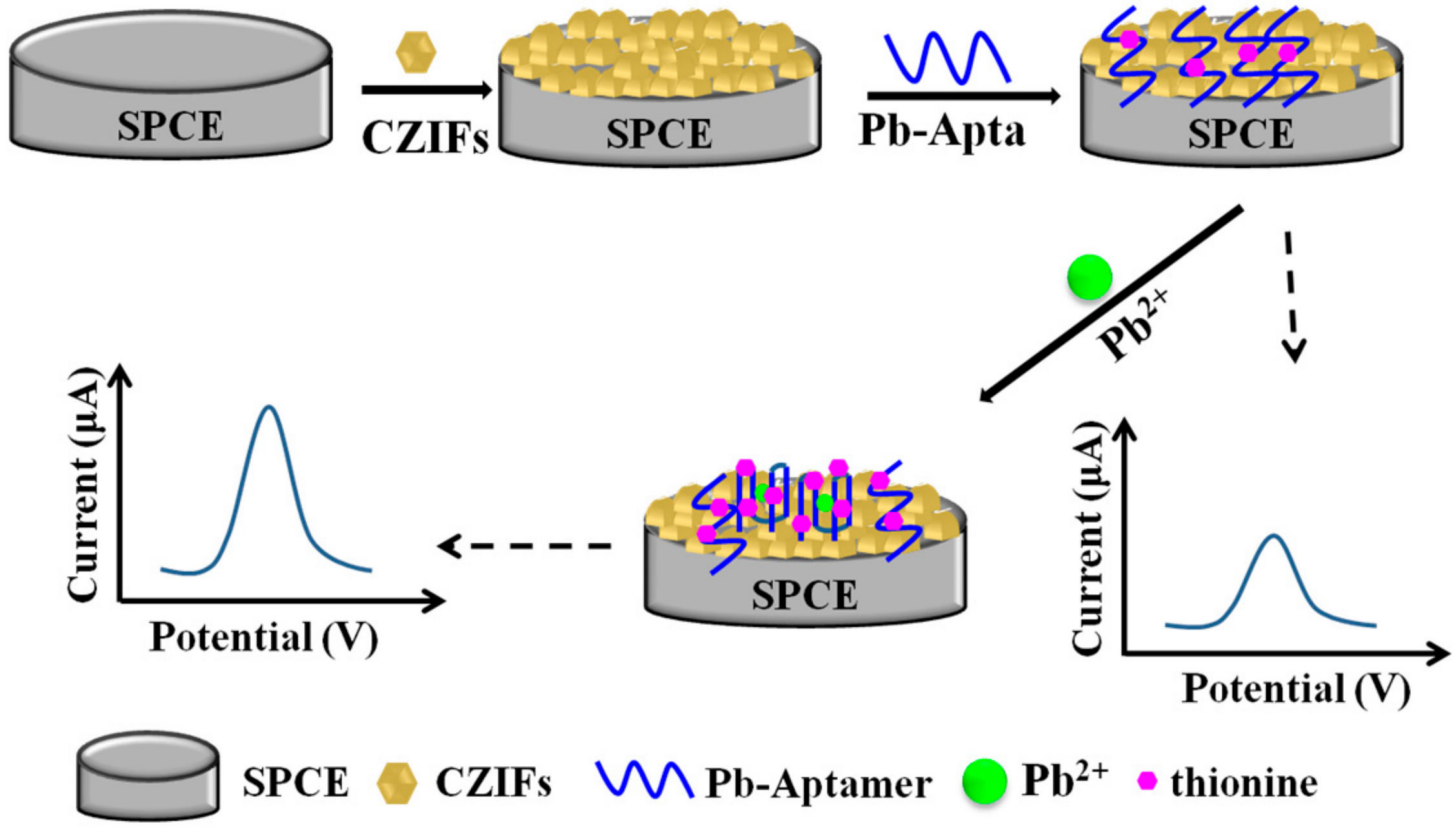
Presentation diagram of the preparation process for the aptasensor and Pb^2+^ detection.

One milligram of CZIFs was dissolved into 1 mL of 1% chitosan solution and ultrasonicated for 5 min. Then, the homogeneous solution was dropped onto the surface of the working electrode and dried at 25 °C. Thereafter, 5.0 μM Pb^2+^ aptamer was evenly introduced on the surface of the working electrode. After it dried, the modified electrodes was rinsed carefully with ultrapure water. Then, the immobilized electrodes were incubated with Pb^2+^ (2 μg L^−1^) for 30 min. After this, the electrodes were rinsed with ultrapure water again. Finally, the cyclic voltammetry (CV) was measured in the HAc-NaAc-thionine buffer of the pH 5.5, scanning from –0.65 V to 0.25 V at a scan rate of 50 mV/s. Differential Pulse Voltammetry (DPV) was performed in 0.1 M HAc-NaAc-thionine scanning in the range of –0.5 to –0.1 V at 50 mV amplitude and 0.5 s pulse width. The preparation process for electrochemical aptasensor and the detecting Pb^2+^ are shown in Scheme 1.

### 2.4. Application in aqueous samples

The river water was collected from Huangpu River, and some cations were added to detect the recovery of the aptasensor. The collection and pretreatment of polluted soil was the same with another paper [40].

## 3. Results and Discussion

### 3.1. Characterization of Porous Carbon Materials

**Figure 1:**
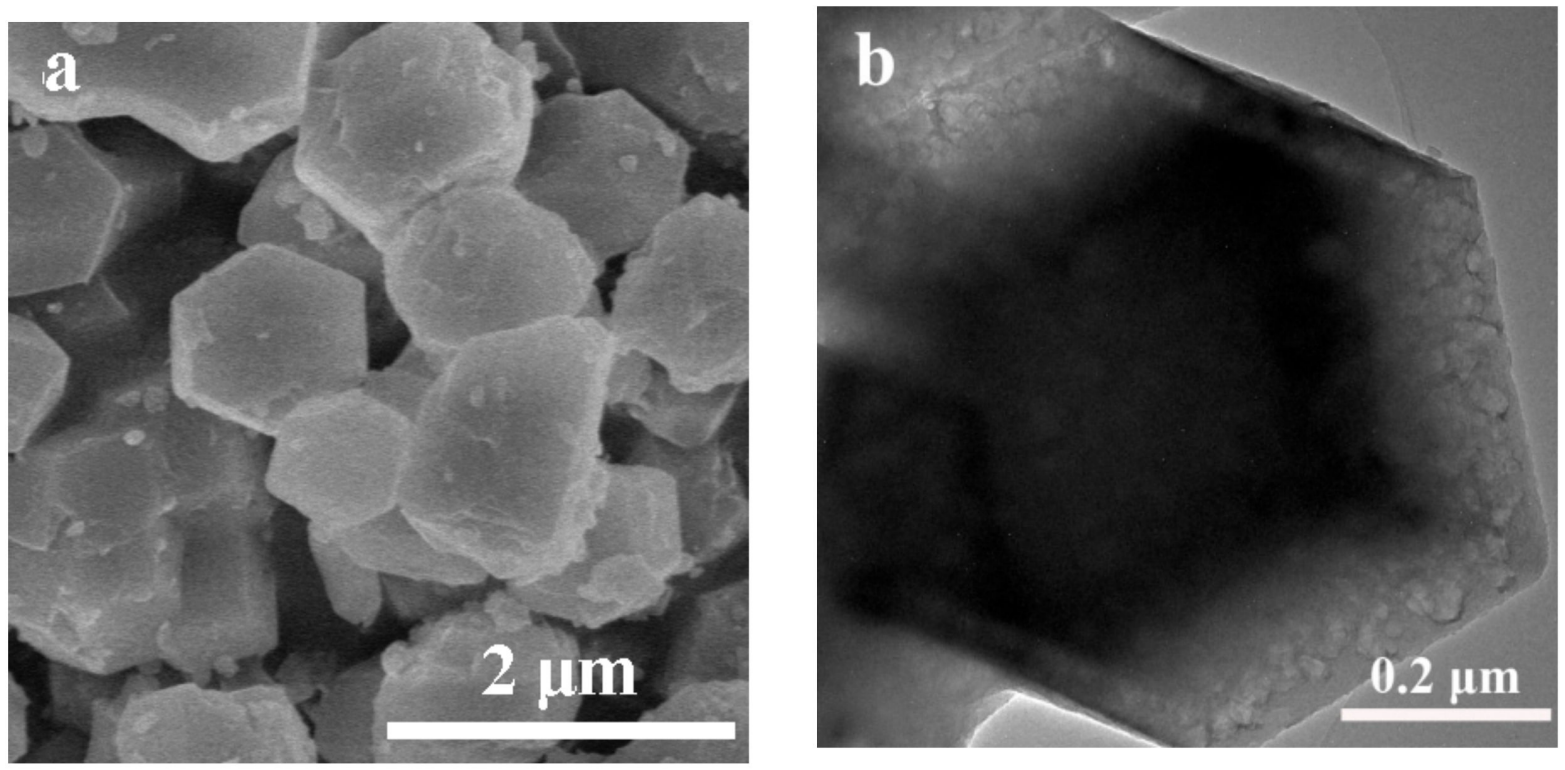
(**a**) SEM image and (**b**) TEM image of ZIF-8@ZIF-67.

The uniform polyhedral nanocrystals of ZIF-8 and ZIF-67 were synthesized. As shown in Figure S1, the volume of ZIF-8 nanocrystals are smaller than ZIF-67 so that the ZIF-8 can be used as the cores for epitaxial growth of ZIF-67. The SEM image of ZIF-8@ZIF-67 presented that the sample displayed entirely uniform rhombic with a smooth surface and the average edge length of the particles is about 420 nm (Figure 1). 

**Figure 2:**
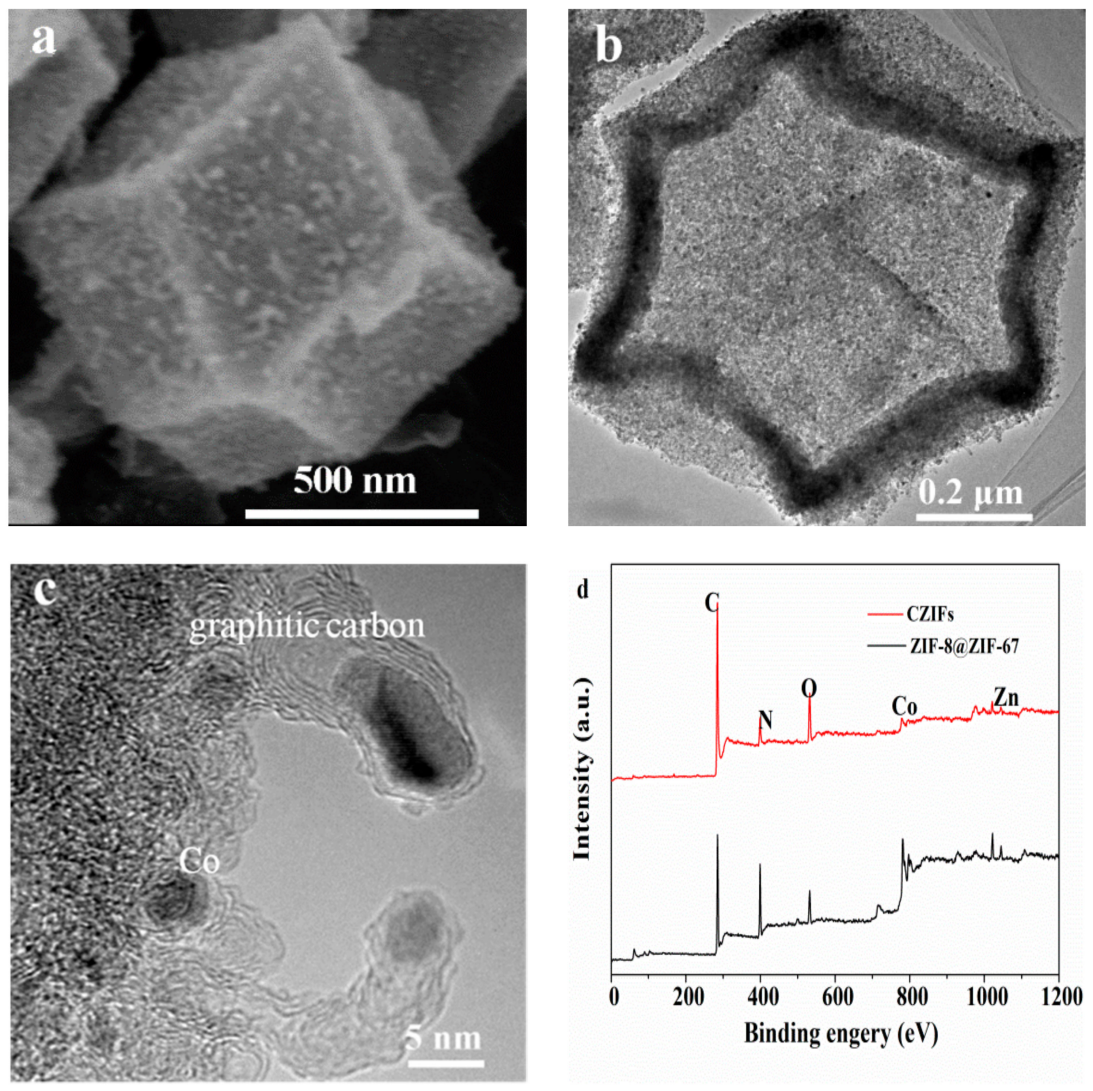
(**a**) SEM image and (**b**,**c**) TEM images of porous carbon, (**d**) XPS full scan of ZIF-8@ZIF-67 and CZIFs.

As shown in Figure 2, the core–shell-structure particles of ZIF-8@ZIF-67 become Metal–Organic-Framework-Derived Hybrid Carbon (CZIFs) by thermal annealing. The SEM image indicated that the CZIFs samples retained crystallite shapes similar to the ZIF-8@ZIF-67 precursors, but the particles have a rough surface anchored with amounts of short carbon nanotubes (Figure 2a,b). Moreover, the high-temperature carbonization process also led to a bit of shrinkage and disintegration. The average edge length is about 420 nm and the outer shells consist of relatively loose carbon with an average thickness of around 70 nm (Figure 2b,c). The formation of short carbon nanotubes is due to the catalytic effect of Co nanoparticles on the particle surface [41]. The image of TEM presented the formation of graphitic carbon structure was decorated with Co nanoparticles and was enclosed in several graphitic carbons with a length of 5 to 15 nm (Figure 2c). The Co nanoparticles in graphitic carbon sheath are not only very stable but can also enhance the stability [42,43]. Since ZIF-67 is less stable than ZIF-8, it would first resolve to form rigid shells of CoO and carbon composites which can generate an outward adhesive force at the interface when the temperature is above 500 °C. The adhesive force is important for keeping the structure of ZIFs because it can prevent the collapse caused by the ZIF-8 induced polycondensation. Then with temperature rise, the outer shells of CoO was reduced to metallic Co with the surrounding carbonaceous become to graphitic carbon [38]. The XPS of CZIFs revealed that the main elementals of the nanoparticles are C, N, O, Co, Zn. Moreover, compared to ZIF-8@ZIF-6, the content of Co and Zn was reduced. One reason is that the unstable Co was washed by diluted sulphuric acid [39]. Another reason is, with the temperature rise, the ZIF-8 decomposed to ZnO and carbon composite and subsequent reduction to Zn and carbon composite when the temperature is above 600 °C, following the Zn to vaporize [42].

### 3.2. Electrochemical Characterization of the Aptasensor

**Figure 3:**
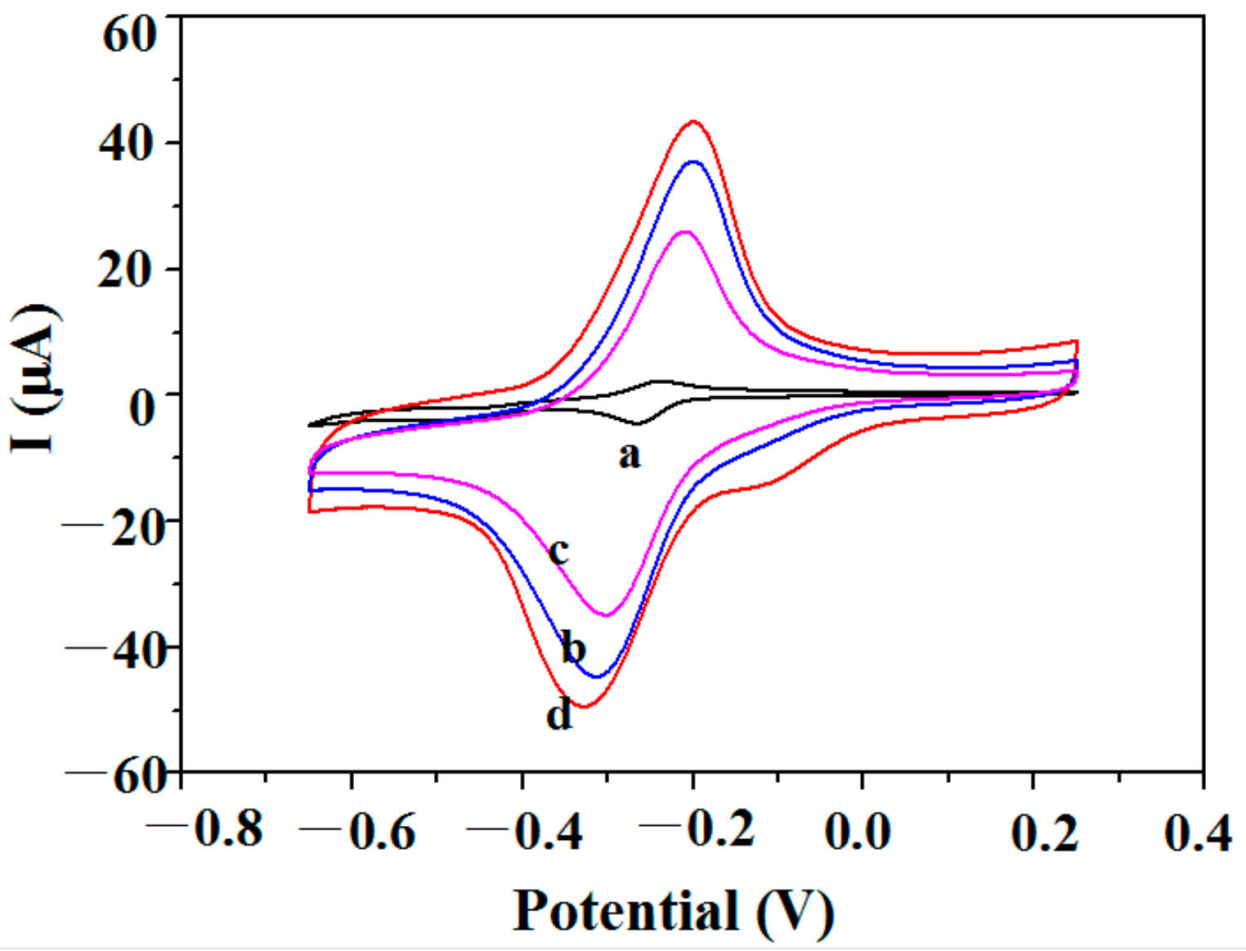
Cyclic voltammetry of different electrodes in testing solution: (a) bare electrode, (b) CZIFs modified electrode, (c) CZIFs/aptamer modified electrode, (d) CZIFs/aptamer/Pb^2+^.

In this aptasensor, the electrochemical behaviors in HAc-NaAc-thionine buffer solution after each modification step were measured by CV (Figure 3). At the bare working electrode, the redox peak of thionine revealed a minimum peak current (Figure 3a), and the peak current increased significantly after the CZIFs were introduced (curve b). The peak current decreased when the working electrode was modified by aptamer (curve c). This phenomenon is that the aptamers are bent, folded, long, and non-conductive oligonucleotide, which can block the electron transfer. In the presence of Pb^2+^, the peak current increased continuously (curve d), suggesting that the aptamer had become the G-quadruplex when Pb^2+^ was introduced. As a substitute, the lead(II)-induced allosteric G-quadruplex oligonucleotide is now being used as a functional DNA molecule for Pb^2+^ sensing [44,45]. It was confirmed that the presence of Pb^2+^ could generate the folding of Aptmer immobilized onto the ERGO/GCE electrode to a G-quadruplex structure [46].

Some reasons can be used to explain the phenomenon. As is known, the aptamer is a bent, folded, long, and non-conductive oligonucleotide. Thionine binds via external stacking to the single strand DNA (ssDNA), and DNA quadruplex only by Coulombic interaction. The electrical conductivity is not affected. In contrast, it intercalates binding to double-strand DNA (dsDNA) base pairs and the electrical conductivity can weaken [47]. In addition, the binding affinities of thionine were remarkably higher with dsDNA compared to ssDNA [48]. Aptamers of Pb^2+^ displayed the double stranded ends, which can bind more thionine, so that the current was decreased with the oligonucleotide introduced. While in the presence of Pb^2+^, the structure of aptamer becomes to G-quadruplex binding thionine by Coulombic interaction which can enhance the electric conductivity. Furthermore, the current was increased with the increase of Pb^2+^. On another hand, some research showed that carbon nanotube could be combined with thionine by π–π reaction which improved the conductive ability [49]. With the aptamer becoming G-quadruplex, a part of nucleic acids left the working electrode so that thionine can be combined with electrode directly [50,51] and accelerated electron transfer under the oxidation and reduction process.

### 3.3. Optimization of Experimental Conditions 

In order to balance the factors of the proposed aptasensor, some experimental parameters affecting the performance, including a concentration of CZIFs and aptamer, a reaction time of aptasensor with Pb^2+^, and pH of buffer solution, were optimized (Figure S4). The results of the optimal experimental conditions are below: concentration of CZIFs: 0.5 mg mL^−1^, (b) concentration of aptamer: 0.5 μM, (c) pH of buffer solution: 5.5, (d) reaction time of Pb^2+^: 40 min. 

### 3.4. Performance of the Aptasensor

**Figure 4:**
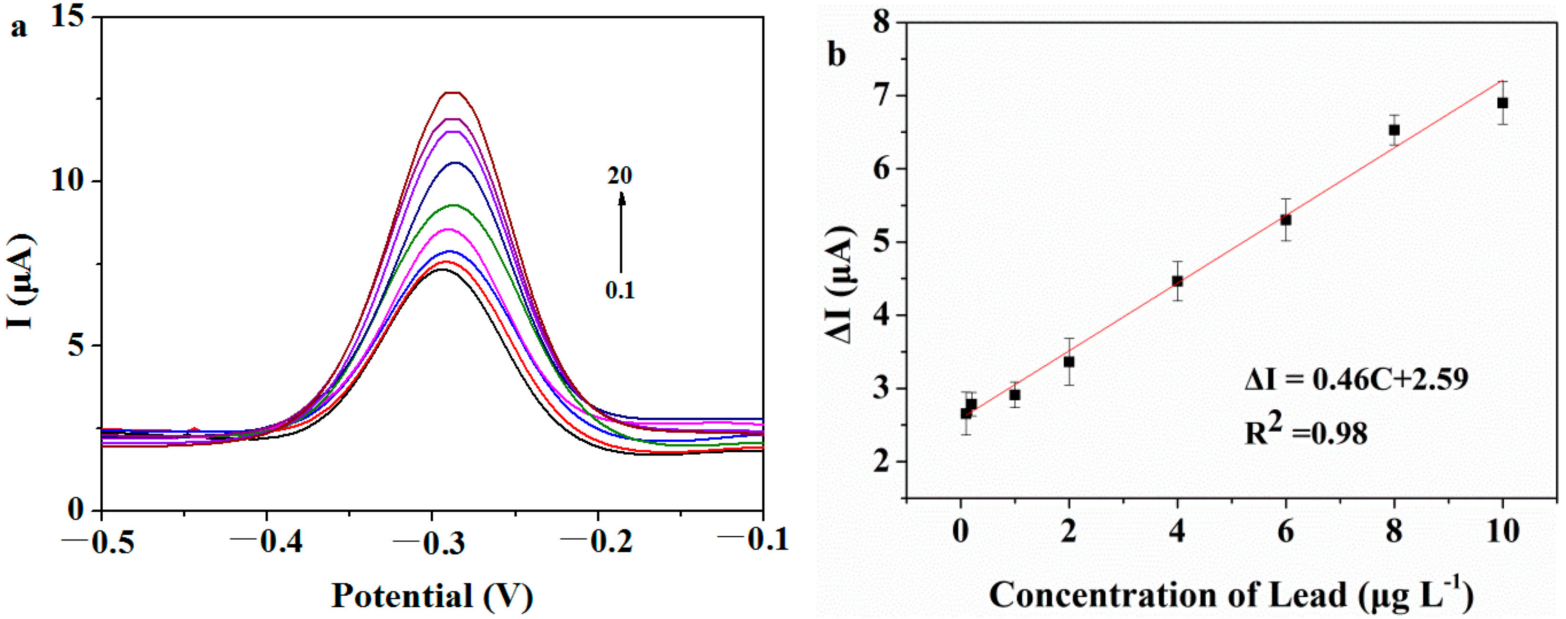
(**a**) Differential Pulse Voltammetry (DPV) responses to aptasensor capturing a series of concentrations of Pb^2+^ from 0.1 to 20 μg L^−1^, (**b**) calibration curve of ΔI and Pb^2+^ concentration.

The DPV was carried out to describe the performance of this aptasensor. Under the most appropriate conditions, difference concentrations of Pb^2+^ were determined. As shown in Figure 4a, the current values increased with the increasing concentration of Pb^2+^. The ΔI increased linearly in the range of 0.1–20 μg L^−1^ of Pb^2+^ and the linear regression equation was ΔI = 0.46C + 2.59 (R^2^ = 0.98, Figure 4b) with the limit of detection (LOD) of 0.096 μg L^−1^ (S/N = 3), where C represents the Pb^2+^ concentration and ΔI is the peak current response (ΔI = Ip’–Ip) before (Ip) and after (Ip’) the target of Pb^2+^ treatment. 

The different methods of range and LOD were compared, and the results are shown in Table 1. Compared with other methods, the LOD of this aptasensor was lower and there are no significant differences with the other methods. However, the detection range of this work still needs to improve. 

### 3.5. Selectivity and Reproducibility of the Aptasensor

**Figure 5:**
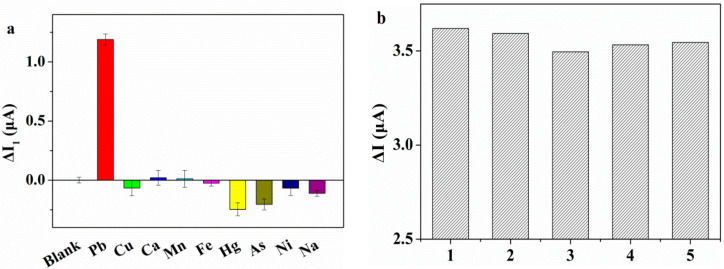
(**a**) Specificity of the aptasensor for Pb^2+^, (**b**) independent detection of the Pb^2+^.

Selectivity is a significant feature for an excellent aptasensor. To evaluate the selectivity of the aptasensor, some heavy metal ions which may interfere with the detection of Pb^2+^ were examined respectively. The concentration of Pb^2+^ was 2 μg L^−1^, the concentrations of Cu^2+^, Ni^2+^, Zn^2+^ were 20 μg L^−1^, and the concentrations of Ag^+^, Mn^2+^, Ca^2+^, Fe^3+^, K^+^, NH_4_^+^ were 10 μg L^−1^. As illustrated in Figure 5a, the other metal cations ΔI’ represented the difference between the ΔI of blank and Pb^2+^ and only changed slightly compared to the blank treatment. As expected, the ΔI’ of Pb^2+^ was higher than other cations, but the high concentrations of K^+^, NH_4_^+^, and Ag^+^ still should be studied in the future.

Moreover, the reproducibility was also significant for the aptasensor. Five equal electrodes were used to invest the reproducibility under the same experimental conditions. As shown in Figure 5b, standard deviation of five independent measurements of Pb^2+^ was 4.04%, indicating that the proposed aptasensor shows good reproducibility.

### 3.6. Assay of Pb^2+^ in River Water and Soil Sample

The application of the proposed assay in actual samples was explored by river water with Pb^2+^ and some other metal cations. The results are shown in Table 2, the mean recoveries were in the range of 96.2~101.5%, and the relative standard deviations (RSD) were below 5.0%.

**Figure 6:**
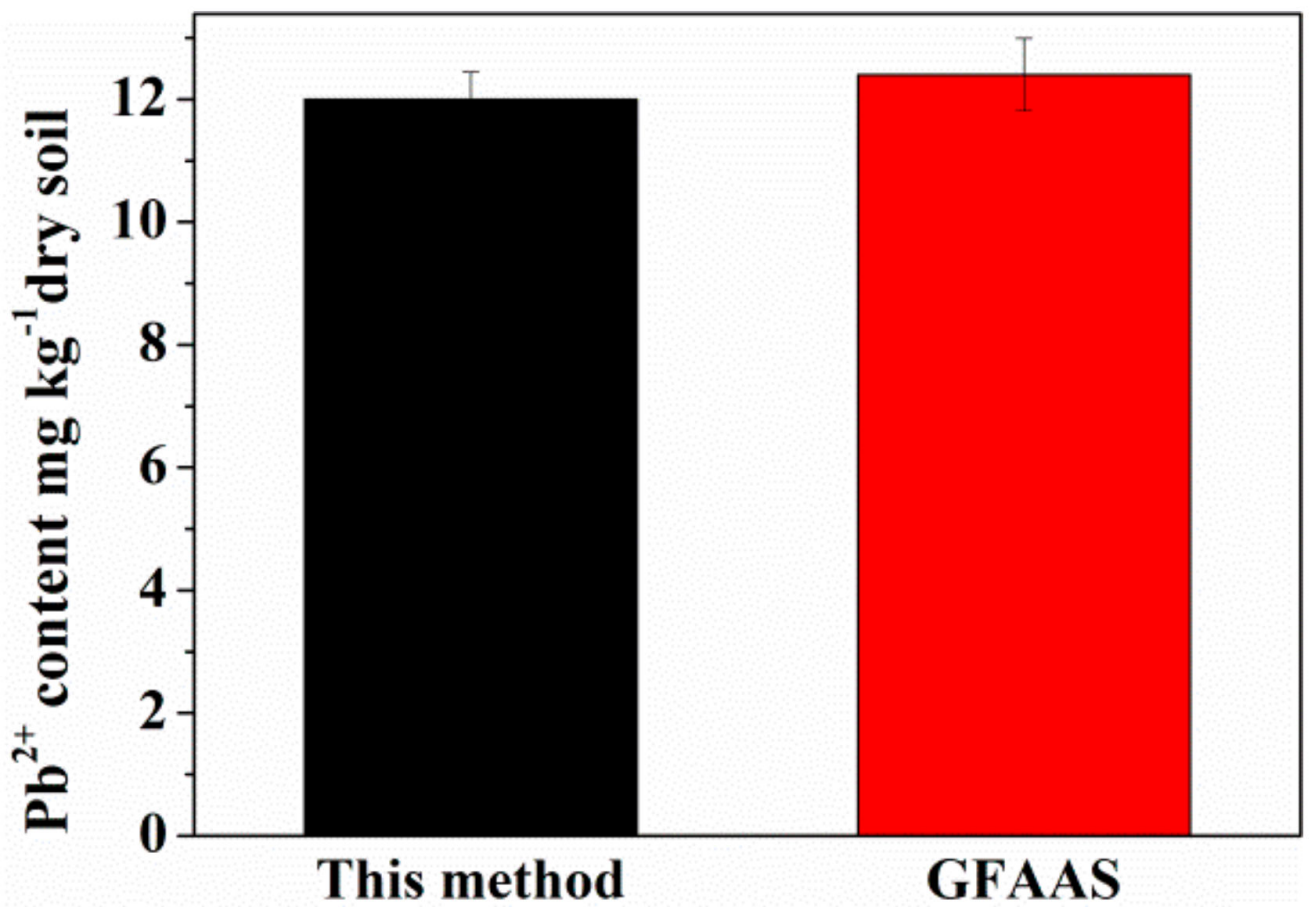
Relative current signal of the polluted soil.

The digested solutions of polluted soils were detected by proposed methods and the presently available method of GFAAS to investigate the feasibility and accuracy of the sensing platform in real samples. As shown in Figure 6, the contents of Pb^2+^ measured by proposed methods and GFAAS were 12.4 mg kg^−1^ and 12.0 mg kg^−1^ in dry soil, respectively. It shows the applicability of the aptasensor as a quantitative method in actual samples.

## 4. Conclusions

In conclusion, an electrochemical aptasensor for rapid and specificity detection of Pb^2+^ was successfully developed. The aptasensor was fabricated after the aptamer was immobilized on SPCE with CZIFs. In the method, CZIFs was used to provide signal transduction and amplification, and the thionine worked as a signal factor. Under optimal conditions, it was demonstrated that the aptasensor could be used to detect Pb^2+^ over a range of concentrations (0.1~10 μg L^−1^) with the LOD of 0.096 μg L^−1^. The method also showed high sensitivity and good selectivity. The feasibility of determining spiked water and polluted soil samples was investigated. This method shows practicality for Pb^2+^ detection in practice. There are some limitations in that K^+^, NH_4_^+^, and Ag^+^ of high concentration may affect the performance of the way, and the work on how to eliminate the effects would be carried out in the future.

## Figures and Tables

**Table 1:** Comparison of different sensors for determination of Pb^2+^.

Method	Linear Range (μg L^−1^)	Detection Limit (μg L^−1^)	Reference
Probe Fe_3_O_4_@Au-ssDNA&Ru-NH2	20.7–20,700	20.7	[52]
The screen-printed electrode (SPE) was immobilized by unique consecutive adenines (polyA) linker and aptamer	0.1–1000	0.03	[53]
Electrochemical aptasensor based on Au@ppy and aptamer	0.1–2.0	0.072	[40]
PDMS-PA-DB_18_C_6_ electrodes were evaluated for anodic stripping voltammetric	20–700	3.5	[54]
Electrochemical aptasensor based on Metal–Organic-Framework-Derived Hybrid Carbon and aptamer	0.1–10	0.096	This work

**Table 2:** Recovery of Pb^2+^ from water samples (n = 4)**.**

**Samples** **(μg L^−1^)**	**Mean Found (μg L^−1^)**	**Mean Recovery (%)**	**RSD (%)**
Pb^2+^ (0.5), Hg^2+^ (2), K^+^ (3.28), Zn^2+^ (15.6), Cu^2+^ (24)	0.48	96.2	4.26
Pb^2+^ (8.0), K^+^ (25.6), Ca^2+^ (50), Mn^2+^ (72.7), Cd^2+^ (17.8)	8.15	101.5	3.95

## Supplementary Materials

The following are available online at https://www.mdpi.com/2079-6374/11/1/1/s1, Figure S1: SEM images of (**a**) ZIF-8 and (**b**) ZIF-67; Figure S2: Optimization of the experimental parameters: (**a**) concentration of porous carbon, (**b**) concentration of aptamer, (**c**) pH of buffer solution, (**d**) different incubation time on aptasensor with Pb^2+.^
