# Supplementary material for: An Electrochemical Aptasensor for Pb2+ Detection Based on Metal–Organic-Framework-Derived Hybrid Carbon
Source: Biosensors (Basel). 2020 Dec 22;11(1):1. doi: 10.3390/bios11010001 (PMC7822124; doi:10.3390/bios11010001)
Supplement: Supplementary file 1 [file biosensors-11-00001-s001.pdf]

biosensors

Supplementary Materials

Article

An Electrochemical Aptasensor for Pb^{2+} Detection Based on Metal–Organic-Framework-Derived Hybrid Carbon

Jina Ding [1,2], Dongwei Zhang [1,2], Yang Liu [1,2], Xuejia Zhan [1,2], Yitong Lu [1,2], Pei Zhou [1,2,]* and Dan Zhang [1,2]

[1] School of Agriculture and Biology, Shanghai Jiaotong University, Shanghai 200240, China; jnding@sjtu.edu.cn (J.D.); Donaghy-Zhang@sjtu.edu.cn (D.Z.); Kimi1201@sjtu.edu.cn (Y.L.); xjzhan@sjtu.edu.cn (X.Z.); ytlu@sjtu.edu.cn (Y.L.); zhdsjtu@sjtu.edu.cn (D.Z.)

[2] Key Laboratory of Urban Agriculture, Ministry of Agriculture and Rural Affairs of the People's Republic of China, Shanghai Jiao Tong University, Shanghai 200240, China

* Correspondence: zhoupei@sjtu.edu.cn; Tel.: 86-02134205762

Received: 26 November 2020; Accepted: 18 December 2020; Published: date

S1 Synthesis of ZIF8 and ZIF67

For the synthesis of ZIF-8, two solutions were first prepared by dissolving 0.894 g of $Zn(NO3)_2{\cdot}6H_2O$ in 30 mL of methanol and 0.984 g of 2-methylimidazole (2-MeIm) in 30 mL of methanol, respectively. Then, the solution of 2-MeIm was quickly poured into the solution of $Zn(NO_3)_2{\cdot}6H_2O$, and the mixture solution was stirred for 24 h at room temperature. Lastly, the solution was centrifuged-washed five times with methanol and dried under vacuum at 60 °C for 12 h. ZIF-67 was synthesized with the same procedure of ZIF-8 except for the use of 0.873 g $Co(NO_3)_2{\cdot}6H_2O$ as metal precursor.

S2 Synthesis of ZIF8@ZIF67 and CZIFs Material

In a typical run, 100 mg of ZIF-8 polyhedrons as the seeds were dispersed in 30 mL of methanol. Then 10 mL, 32.8 $mg{\cdot}mL^{-1}$ methanol solution and 150 mg of $Co(NO_3)_2{\cdot}6H_2O$ were added into the suspension of ZIF-8 polyhedrons under magnetic stirring with 300 rpm. After stirring for 24 h, the ZIF-8@ZIF-67 core-shell particle was centrifuged-washed five times at 8000 rpm with methanol and dried under vacuum at 60 °C for 12 h. The as-prepared ZIF-8@ZIF-67 particles were annealed at 800 °C for 5 h under an argon gas flow with a heating rate of 10 °C/min, and the yielding black powder was moved to a 50 mL centrifuge tube and then added 10 mL 0.5 M H_2SO_4 solution with a water bath at at 80 °C for 12 h to remove the unstable metal species. The black powder was centrifuged with 8000 rpm and washed three times by ultrapure water and then dried under vacuum at 60 °C for 6 h.

S3 SEM Images of ZIF-8 and ZIF-67

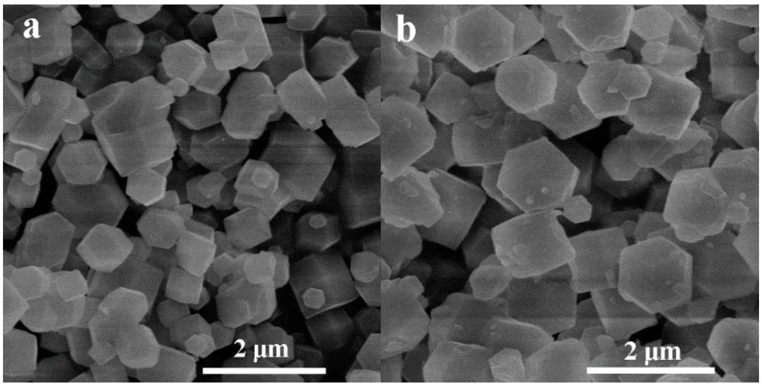

Figure S1. SEM images of (**a**) ZIF-8 and (**b**) ZIF-67.

S4 Optimization of Experimental Conditions

As illustrated in Figure S4a, with the increase of the concentration of CZIFs from 0.25 to 1.0 mg·mL^{-1}, the ΔI reached the maximum at 0.5 mg·mL^{-1}, indicating that 0.5 mg·mL^{-1} of CZIFs was sufficient for the reaction. Therefore, 0.5 mg·mL^{-1} was selected as the suitable concentration of CZIFs material. The different concentrations of aptamer was optimized and the ΔI got the maximum value at 0.5 μM (Figure S4b). Therefore, 5 μM was selected as the suitable aptamer concentration. As shown in Figure S4c, the pH of buffer solution was optimized. With the increased the concentration of pH, the ΔI reached the maximum at 5.5, indicating that pH 5.5 was the most suitable in the below experiments. As shown in Figure S4d, above 40 mins, the ΔI signal almost kept invariable, suggesting that 40 mins was suitable as the incubating time of aptasensor and Pb^{2+}. Hence, the incubating 40 mins was adopted in the following work.

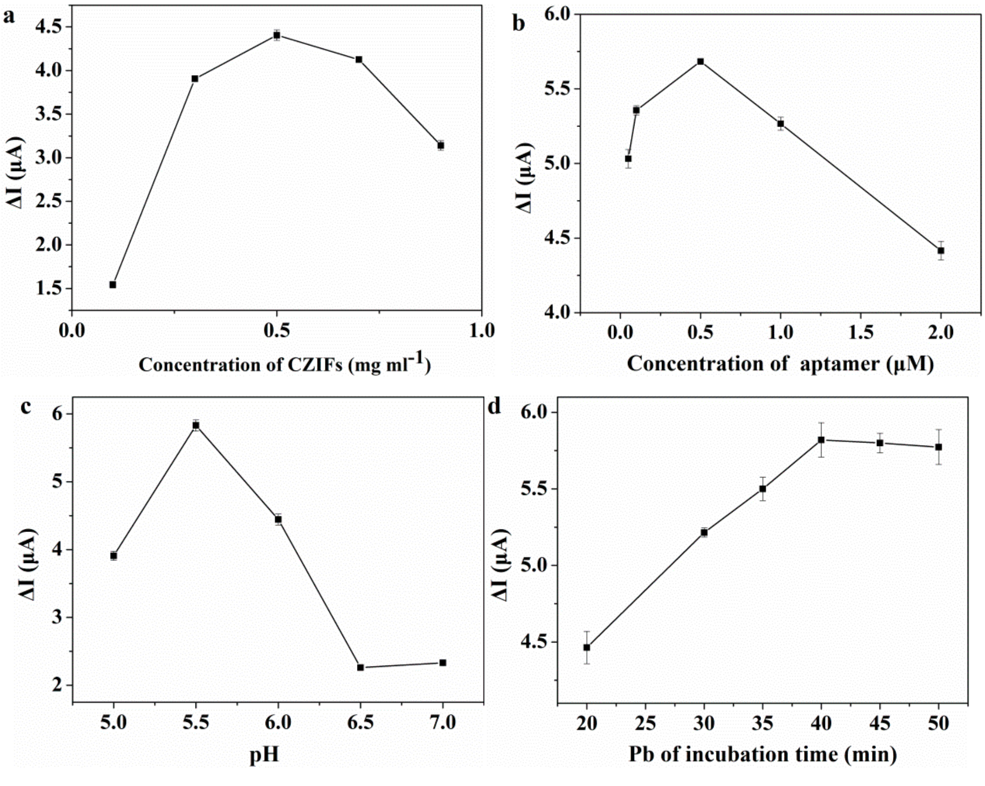

Figure S2. Optimization of the experimental parameters: (**a**) concentration of porous carbon, (**b**) concentration of aptamer, (**c**) pH of buffer solution, (**d**) different incubation time on aptasensor with $Pb^{2+.}$

© 2020 by the authors. Licensee MDPI, Basel, Switzerland. This article is an open access article distributed under the terms and conditions of the Creative Commons Attribution (CC BY) license (http://creativecommons.org/licenses/by/4.0/).
